# Predicting Risk of Hospital Admission in Patients With Suspected COVID-19 in a Community Setting: Protocol for Development and Validation of a Multivariate Risk Prediction Tool

**DOI:** 10.2196/29072

**Published:** 2021-05-25

**Authors:** Ana Belen Espinosa-Gonzalez, Ana Luisa Neves, Francesca Fiorentino, Denys Prociuk, Laiba Husain, Sonny Christian Ramtale, Emma Mi, Ella Mi, Jack Macartney, Sneha N Anand, Julian Sherlock, Kavitha Saravanakumar, Erik Mayer, Simon de Lusignan, Trisha Greenhalgh, Brendan C Delaney

**Affiliations:** 1 Department of Surgery and Cancer Imperial College London London United Kingdom; 2 Patient Safety Translational Research Centre, Institute of Global Health Innovation Imperial College London London United Kingdom; 3 Center for Health Technology and Services Research / Department of Community Medicine, Health Information and Decision (CINTESIS/MEDCIDS) Faculty of Medicine, University of Porto Porto Portugal; 4 Nuffield Department of Primary Care Health Sciences University of Oxford Oxford United Kingdom; 5 Whole Systems Integrated Care, North West London Clinical Commissioning Group London United Kingdom

**Keywords:** COVID-19 severity, risk prediction tool, early warning score, hospital admission, primary care, electronic health records

## Abstract

**Background:**

During the pandemic, remote consultations have become the norm for assessing patients with signs and symptoms of COVID-19 to decrease the risk of transmission. This has intensified the clinical uncertainty already experienced by primary care clinicians when assessing patients with suspected COVID-19 and has prompted the use of risk prediction scores, such as the National Early Warning Score (NEWS2), to assess severity and guide treatment. However, the risk prediction tools available have not been validated in a community setting and are not designed to capture the idiosyncrasies of COVID-19 infection.

**Objective:**

The objective of this study is to produce a multivariate risk prediction tool, RECAP-V1 (Remote COVID-19 Assessment in Primary Care), to support primary care clinicians in the identification of those patients with COVID-19 that are at higher risk of deterioration and facilitate the early escalation of their treatment with the aim of improving patient outcomes.

**Methods:**

The study follows a prospective cohort observational design, whereby patients presenting in primary care with signs and symptoms suggestive of COVID-19 will be followed and their data linked to hospital outcomes (hospital admission and death). Data collection will be carried out by primary care clinicians in four arms: North West London Clinical Commissioning Groups (NWL CCGs), Oxford-Royal College of General Practitioners (RCGP) Research and Surveillance Centre (RSC), Covid Clinical Assessment Service (CCAS), and South East London CCGs (Doctaly platform). The study involves the use of an electronic template that incorporates a list of items (known as RECAP-V0) thought to be associated with disease outcome according to previous qualitative work. Data collected will be linked to patient outcomes in highly secure environments. We will then use multivariate logistic regression analyses for model development and validation.

**Results:**

Recruitment of participants started in October 2020. Initially, only the NWL CCGs and RCGP RSC arms were active. As of March 24, 2021, we have recruited a combined sample of 3827 participants in these two arms. CCAS and Doctaly joined the study in February 2021, with CCAS starting the recruitment process on March 15, 2021. The first part of the analysis (RECAP-V1 model development) is planned to start in April 2021 using the first half of the NWL CCGs and RCGP RSC combined data set. Posteriorly, the model will be validated with the rest of the NWL CCGs and RCGP RSC data as well as the CCAS and Doctaly data sets. The study was approved by the Research Ethics Committee on May 27, 2020 (Integrated Research Application System number: 283024, Research Ethics Committee reference number: 20/NW/0266) and badged as National Institute of Health Research Urgent Public Health Study on October 14, 2020.

**Conclusions:**

We believe the validated RECAP-V1 early warning score will be a valuable tool for the assessment of severity in patients with suspected COVID-19 in the community, either in face-to-face or remote consultations, and will facilitate the timely escalation of treatment with the potential to improve patient outcomes.

**Trial Registration:**

ISRCTN registry ISRCTN13953727; https://www.isrctn.com/ISRCTN13953727

**International Registered Report Identifier (IRRID):**

DERR1-10.2196/29072

## Introduction

### Overview

During 2020, it became clear that assessment of the severity of COVID-19 infection required clinical tools specific to the condition and that repurposing tools such as the National Early Warning Score (NEWS2), designed for the early diagnosis of sepsis, would not be safe clinical practice [1]. The management of COVID-19 by clinicians is challenged by uncertainty about the disease progression [2]. There is evidence that a small percentage of patients present a dramatic deterioration of clinical status around the 8th to 10th day of disease, often associated with unperceived low oxygen saturations (known as “silent hypoxia”) that may require hospital and intensive care unit (ICU) admissions [3-5]. The inability to predict which patients will experience clinical deterioration adds an additional level of complexity to the clinical challenge and diagnostic uncertainty that general practitioners (GPs) have faced during the pandemic, particularly as most of the consultations are carried out remotely (usually by telephone and occasionally by video) to minimize the risk of transmission [6].

It was initially suggested that NEWS2 could be used to assess severity of patients with COVID-19 [7]. NEWS2 is calculated from patient’s temperature, pulse rate, respiratory rate, systolic blood pressure, pulse oximetry reading, and presence of new onset of acute confusion [8]. It is commonly used in hospital settings and ambulance service prior to transfer to hospital to assess the risk of deterioration of a patient [9]. However, NEWS2 seems to be a late indicator of decompensation, typically triggering within the last 12 hours before a transfer to ICU is considered necessary and, therefore, this limits its application and validity in a primary care or community care setting where an earlier warning would be preferred [9,10].

The Roth score (originally developed as a measure of breathlessness in cardiopulmonary disease [11]) was briefly considered by the Royal College of General Practitioners (RCGP) as possibly useful in the assessment of breathlessness when assessing patients with signs and symptoms of COVID-19 [12]. However, a rapid literature review concluded that the Roth score might have a low sensitivity (ie, a normal score in patients with “silent hypoxia”), and therefore should not be used by GPs when assessing patients over the phone or in video consultations [13].

### Justification and Study Objective

This new condition and the forced shift toward remote consultations during the pandemic have increased the challenges and uncertainty commonly faced in general practice [6]. Primary care clinicians need a tool to guide the management of patients with suspected COVID-19 to be able to identify those whom they can reassure, those that need monitoring, and those that require urgent further assessment or referral to hospital. Even though the validity of NEWS2 for this purpose was a subject of intense debate during the height of the first COVID-19 wave, the score is still being used by primary care clinicians to assess patients prior to transfer to hospital [9]. The use of NEWS2 outside the hospital setting has not been validated, and it was not designed to capture the idiosyncrasies of COVID-19 infection. Therefore, there is need to develop an early warning score that incorporates key features of acute COVID-19 and that can be safely used by GPs when assessing patients remotely [14].

We reviewed the literature on COVID-19 early warning scores, then conducted a series of focus groups with 72 primary care clinicians (mostly GPs and including advanced nurse practitioners and paramedics) to derive elements that might form part of a suitable score, value sets, and appropriate SNOMED terms [15]. This paper describes the process of quantitative development and validation of the Remote COVID-19 Assessment in Primary Care (RECAP) score. The objective was to produce a multivariate risk prediction tool to facilitate the early identification, by primary care physicians and other clinicians working in the community, of those patients with COVID-19 that are at higher risk of becoming severely ill and inform the early escalation of their treatment, while also reducing unnecessary referrals in low-risk patients, with the aim of improving patient outcomes.

## Methods

### Study Design

**Figure 1:**
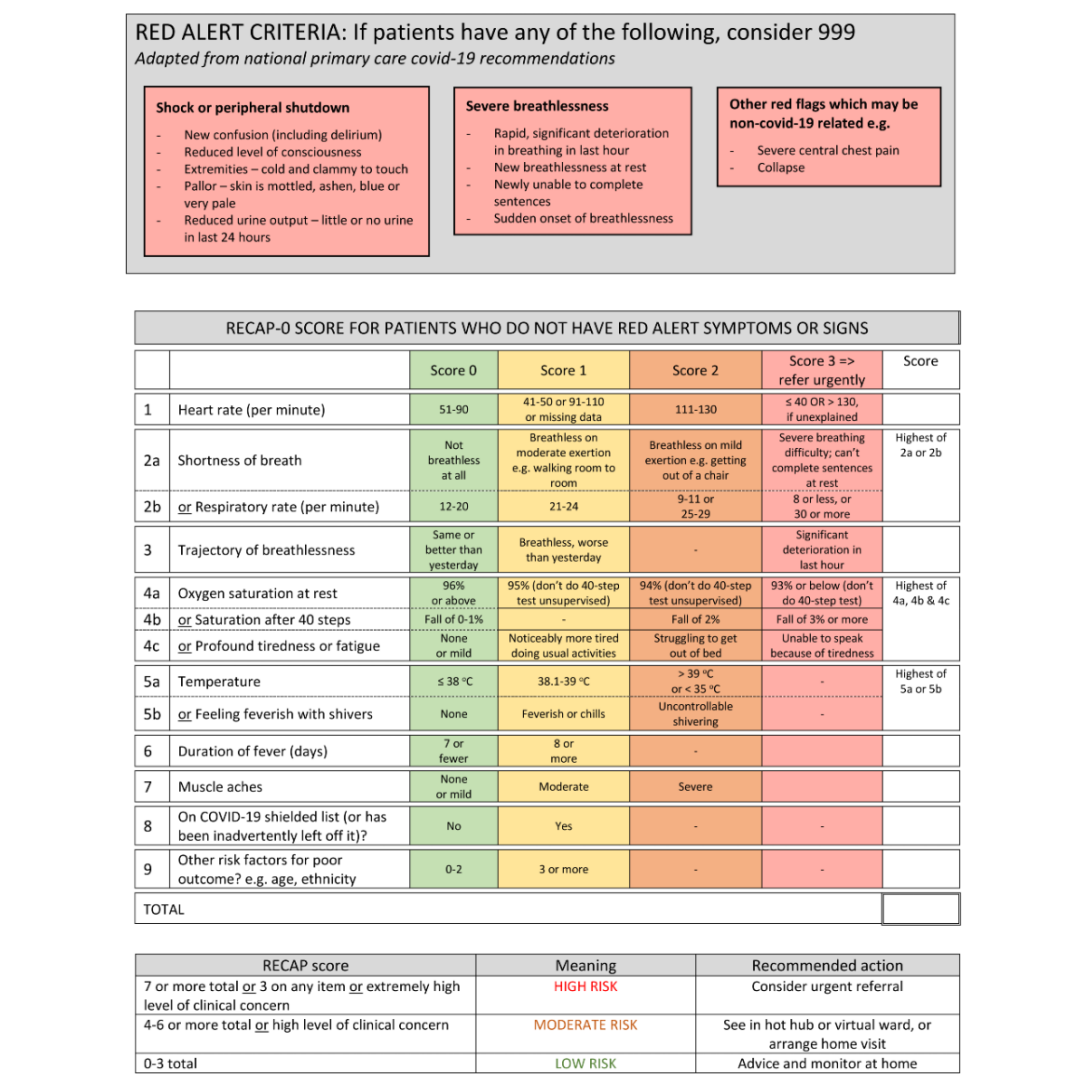
Summary of RECAP-V0 items. Source: [15]. RECAP: Remote COVID-19 Assessment in Primary Care.

This primary care data linkage study follows a prospective cohort observational design, whereby patients presenting in primary or community care with signs and symptoms suggestive of COVID-19 will be followed and their data linked with hospital outcomes, particularly focusing on hospital admission, ICU admission, and death. For data collection purposes, the initial set of items identified in earlier qualitative work [15], known as RECAP-V0, will be integrated into an electronic template to be used by primary care physicians (see Figure 1 for a summary of items included in RECAP-V0). This will enable the standardized recording of patients’ signs and symptoms and subsequent linkage with hospital and mortality data. Data collected will be used to develop and validate a multivariate regression model to predict hospital admission, ICU admission, and death.

### Data Collection

#### Recruitment

The development of the RECAP score will require the use of primary and secondary data. The collection of patients’ signs and symptoms as they present in primary care requires the involvement of primary care clinicians, who will be asked to assess those patients with a clinical diagnosis of suspected COVID-19 using the RECAP electronic template.

The recruitment of clinicians (study sites) and patients (study participants) will be carried out by four different arms depending on clinician and participant location and service used to seek medical care:

1. North West London (NWL) Clinical Commissioning Groups (CCGs) arm: this arm has its own integrated linked database (Whole Systems Integrated Care [WSIC]) and a secure environment (Imperial’s Clinical Analytics, Research and Evaluation [iCARE] secure environment) to hold the data. Recruitment of practices will be facilitated by the NWL clinical research network (CRN). General practitioners will use EMIS [16] or TPP SystmOne [17] electronic health record systems to capture patients’ data.
2. RCGP Research and Surveillance Centre (RSC) arm: this is a national network of practices within the RCGP developed to contribute with data for disease surveillance and research [18], which is held in the Oxford RCGP Clinical Informatics Digital Hub (ORCHID) secure environment [19]. Subject to the patient’s consent, data from RSC network practices (collected from computerized medical record systems EMIS or TPP SystmOne, the United Kingdom’s most used systems, using Ardens RECAP electronic templates [20]) will be pseudonymized and extracted via a Wellbeing Software extraction system and linked to outcomes.
3. Covid Clinical Assessment Service (CCAS) arm: this service is organized within the National Health Service (NHS) 111 Online service (managed by South Central Ambulance Service) for the clinical assessment and management of patients with a clinical diagnosis of suspected COVID-19. It is staffed by general practitioners and uses the Adastra electronic health record system [21]. Upon patients’ consent, the data collected will be transferred to ORCHID and linked to hospital outcomes.
4. Doctaly arm: this private health care platform has been commissioned by South East London CCGs to provide a home monitoring service for patients with a diagnosis of COVID-19 (positive result in laboratory test) in South East London. Patients’ medical history and assessment data are collected using a chatbot via the WhatsApp mobile app. The questions asked via the Doctaly chatbot were designed to reflect the same concepts as the RECAP-V0 set. Data collected will be also transferred to the Oxford secure environment and linked to outcome data.

**Figure 2:**
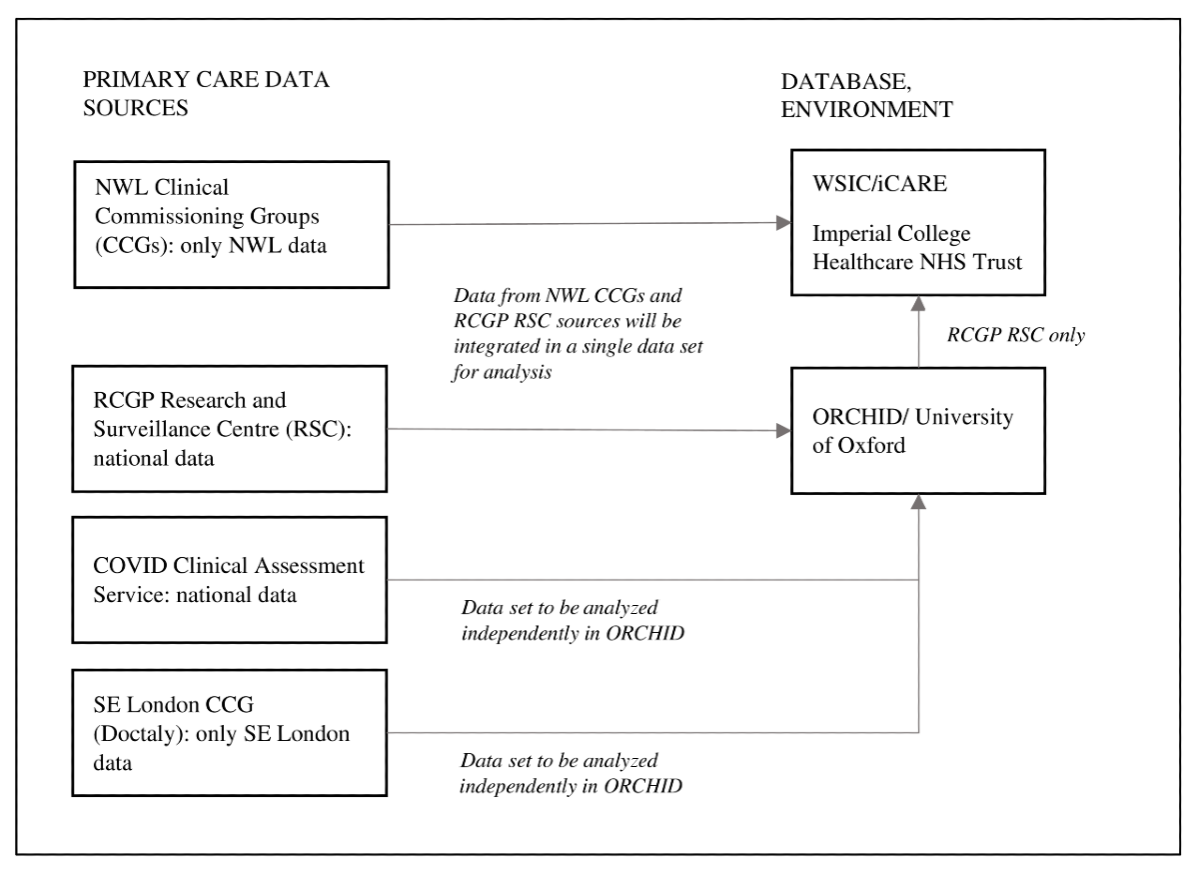
Data flowchart. CCG: Clinical Commissioning Group; NHS: National Health Service; NWL: North West London; ORCHID: Oxford RCGP Clinical Informatics Digital Hub; RCGP: Royal College of General Practitioners; RSC: Research and Surveillance Centre; SE: South East.

Figure 2 below depicts study data sources and data flow. Primary care data collected by practices in NWL and held in iCARE are already linked to hospital outcomes (ie, hospital admission, ICU admission, and death). Data held in the University of Oxford secure environment (RCGP RSC, CCAS, and Doctaly data) will be linked to outcome data contained in the Hospital Episode Statistics (HES) and Office of National Statistics (ONS) databases using an encrypted NHS number. Hospital admission and mortality data are available in HES and ONS; however, ICU admission information is not available.

#### Selection Criteria

Our main cohort includes patients clinically diagnosed with COVID-19 that are being assessed and managed in primary care. Additional cohorts include patients with signs and symptoms suggestive of COVID-19 assessed by the NHS 111 CCAS and patients with established COVID-19 that are assessed as part of a primary care–led home monitoring service (Doctaly).

In the NWL, RCGP RSC, and CCAS arms, participants will be identified by primary care clinicians and enrolled in the study if they satisfy the following inclusion criteria:

1. The patient is willing and able to provide informed consent for data linkage (exceptions are described in detail in the Overview section of the Results)
2. The patient has signs and symptoms that are judged by the clinician to be suggestive of acute COVID-19 and time since onset of symptoms is ≤14 days.
3. The participant is 18 years of age or older.
4. The clinician is able to use the electronic template that contains the RECAP codes.
5. Data collected by the clinician can be linked to the following hospital outcomes: hospital admission, ICU admission (only for NWL CCGs arm data), and hospital outcome (either discharge or cause of death).

For data collected in South East London CCGs (Doctaly) arm, the selection criteria consist of participant age (ie, 18 years old or older) and having a data sharing or consent procedure in place, since the other criteria are already satisfied (ie, patients are offered home monitoring after receiving a positive result from a COVID-19 test and the monitoring tool was specifically designed to include RECAP codes).

#### Template Development

In order to collect primary data from primary care or community care settings, the RECAP-V0 items that captured patients’ signs and symptoms along with other characteristics (sociodemographic information and comorbidities) are transferred into an electronic template using SNOMED and Read codes. These codes have been identified by the study team and collaborators and have been reviewed by NHSX, NHS England, and the UK Faculty of Clinical Informatics. The templates have been deployed for COVID-19 management via electronic health record systems—such as Ardens EMIS and SystmOne, TPP SystmOne, or Adastra—used by clinicians in GP practices, COVID-19 hubs, and CCAS, or via the patient-facing platform Doctaly. This will enable the collection of patients’ signs and symptoms in large data sets that will be stored in two secure environments (ORCHID and iCARE secure environments).

### Sample Size

A total of 2880 participants will be necessary to develop a model with a minimum 85% specificity, assuming 10% prevalence of hospital admission and 6% missing data. We will split the sample into two consecutive groups, taking the first 50% of participants’ data for model development and the last 50% for model validation. CCAS will also collect 2880 participants as we wish to explore the hypothesis that, on account of case mix and spectrum bias, patients already triaged to the national service may require a separate model. We will then separately develop and validate a model for CCAS. Doctaly will provide an additional validation data set for RECAP-V1 score.

### Data Analysis

#### Overview

A detailed statistical analysis plan (SAP) written before inspecting the data will be followed for analysis. The SAP provides a detailed description of data handling, RECAP-V1 model development and validation, and any planned secondary outcomes analysis. Given the complexity of issues to be addressed, including missing data not at random, potential correlations between clinical measurements; regression models and machine learning; and the relationships between the four different data sets, the SAP will be the subject of a separate article.

#### RECAP-V1 Early Warning Score Development and Validation

We will use multivariate logistic regressions to develop and validate the score. Table 1 contains a list of the items we included in the RECAP-V0 electronic template along with their SNOMED codes that will be used as inputs in the model.

The template has been designed to support the assessment of patients via both face-to-face and remote consultations; however, we anticipate that there are certain observations, such as respiratory rate or oxygen saturation, whose recording in remote consultations may be challenging. Therefore, we included information on patients’ symptoms that could be used as a proxy of quantitative items if they were unavailable. The factors for the model (predictor variables) can then be summarized as follows: heart rate, respiratory rate or shortness of breath, trajectory of breathlessness, oxygen saturation or level of tiredness, temperature or feeling feverish, days from onset of symptoms, muscle aches, and cognitive decline. Moreover, we will extract other patient characteristics such as age, gender, body mass index, ethnicity, presence of comorbidities (eg, diabetes, hypertension, coronary heart disease, and chronic kidney disease), and whether the patient is or has been on a COVID-19 shielding list. During the conduct of the study, the QCOVID score [22] has been adopted as a measure of baseline risk and used to populate the COVID-19 shielding list in health record systems [23]. We expect that patient characteristics ought to be able to be represented by the shielding term and will test this hypothesis. Missing data will be handled with standard methodologies for the multiple imputation of missing data [24].

Regarding the outputs of the model, we are interested in hospital admission (defined as an overnight hospital stay within 28 days of onset of symptoms), ICU admission (only available in NWL’s WSIC/iCARE database), and death (either at the hospital or at home within 28 days of onset of symptoms).

We will also conduct exploratory analyses, using machine learning algorithms for outcome prediction (nonlinear classifiers) including random forest, gradient boosting, and neural networks, alongside machine learning approaches for imputation of missing data.

**Table 1:** RECAP-V0 template items.

Variable name	Measurement level	Description/parameter	Concept ID
Heart rate	Continuous	Heart rate measured at systemic artery (observable entity)	78564009
Respiratory Rate	Continuous	Respiratory rate (observable entity)	86290005
Shortness of breath	Nominal	No breathlessness (situation) Breathless, moderate exertion (finding) Breathless, mild exertion (finding) Unable to complete a sentence in one breath (finding)	161938003 161939006 161940008 407588003
Trajectory of breathlessness	Nominal	Patient condition improved (finding) Patient condition unchanged (finding) Patient condition deteriorating (finding) Symptom very severe (finding)	268910001 359748005 275723000 162471005
Oxygen saturation (rest)	Continuous	Peripheral blood oxygen saturation on room air at rest (observable entity)	866661000000106
Oxygen saturation (exertion)	Continuous	Peripheral blood oxygen saturation on room air on exertion (observable entity)	866681000000102
Level of tiredness	Nominal	Not tired (situation) Fatigue (finding) Unable to get on and off a bed (finding)	161869003 84229001 301663005
Temperature	Continuous	Tympanic temperature (observable entity)	703421000
Feeling feverish	Nominal	No temperature (situation) Feeling hot (finding) Rigor symptom (finding)	161851007 373904004 248457000
Date of onset of symptoms	Continuous	Date of onset of symptoms (observable entity)	520191000000103
Muscle aches	Nominal	Myalgia (finding)	68962001
Cognitive decline	Nominal	Mentally alert (finding) Clouded consciousness (finding) Acute confusion (finding) On examination, decreased level of consciousness (finding)	248234008 40917007 130987000 417473004
COVID-19 shielding list as defined using the QCOVID score [23]	Nominal	High-risk category for developing complication from COVID-19 infection (finding)	1300561000000107
Age	Continuous	Current chronological age (observable entity)	424144002
Body mass index	Continuous	Body mass index (observable entity)	60621009
Patient sex	Nominal	Patient sex (observable entity)	184100006
Diabetes	Nominal	Diabetes (disorder)	73211009
Hypertension	Nominal	Hypertension (disorder)	38341003
Coronary heart disease	Nominal	Coronary heart disease (disorder)	53741008
Chronic kidney disease	Nominal	Chronic kidney disease (disorder)	709044004
Ethnicity/related nationality data	Nominal	Ethnicity/related nationality data (observable entity)	186034007
Participant’s consent	Nominal	Consent given to participate in research study (finding)	873771000000107

## Results

### Overview

Recruitment started in October 2020. Initially, only the NWL CCGs and RCGP RSC arms were actively recruiting. In order to engage clinicians with the study and facilitate participation, we have run three webinars or training workshops where the study team described the study objectives and deadlines, and provided a detailed description of the RECAP template and how it would be used at the clinical front line. These webinars took place in October 2020 and January 2021. The study team has been in close contact with participating practices through the Imperial College London arm office, which has overall responsibility for the project and is directly in charge of data collection in North West London, and the University of Oxford arm office, which has direct responsibility for data collection from RCGP RSC practices. The CCAS and Doctaly platform arms joined the study in February 2021.

The initial data set to be used for model development will consist of RCGP RSC and NWL arms data and will be complete by the end of March 2021; this includes the primary care data on recruited patients’ signs and symptoms collected by these two arms linked to outcomes 28 days later. Two stages of data extraction and analysis of this integrated data set have been identified: first, RECAP-V1 development using the first half of the data set will start in April 2021, and second, model validation using the second half of the data set will follow. As of March 24, 2021, we have recruited a combined sample of 3827 participants (173 active primary care practices enrolled) in these two arms. The CCAS arm started the recruitment process on March 15, 2021, and we expect to reach the desired sample size in this arm (2880 participants) by the end of May 2021. Data sharing agreements are being developed to access data that have already been collected from around 1400 participants by clinicians using the Doctaly platform. The CCAS and Doctaly platform data sets will be used to validate the RECAP-V1 model and will be analyzed independently. Once we have produced the model, and subject to findings, the RECAP-V1 score will be ready to be deployed and used by clinicians to guide the management of patients with suspected COVID-19 according to their predicted risk.

The study is sponsored by Imperial College London and ethical approval was granted by the North West-Greater Manchester East Research Ethics Committee and Health Research Authority on May 27, 2020 (Integrated Research Application System number: 283024, Research Ethics Committee reference number: 20/NW/0266). An amendment to include the CCAS and Doctaly arms was approved on February 1, 2021. Due to the low risk associated with participation in this study and the remote nature (telephone/video consultation) of most patient encounters, the review committee agreed that obtaining verbal consent for data linkage was acceptable.

To access and link retrospective data collected by the NHS 111 CCAS and Doctaly platforms in South East London (ie, data that have already been collected by the services prior to study participation) we requested the last ethics amendment submitted to be assessed under the Control of Patient Information (COPI) notice, data sharing provisions that allow public authorities and research bodies the use of COVID-19–relevant patient-level data without the need for patients’ explicit consent [25]. For NHS 111 CCAS prospective data—that is, data from patients seeking medical care after the RECAP template has been installed in Adastra—we will apply the same mechanism to seek consent that has been followed in the NWL and RCGP RSC arms, and patients in the clinical queue will receive an SMS text message with information on the study and how to participate.

Data and all appropriate documentation will be stored in accordance with General Data Protection Regulation (Data Protection Act 2018) for a minimum of 10 years after the completion of the study, including the follow-up period. Participants can withdraw from the study at any point by informing their GP or a member of the study team. They will be asked whether the data obtained before withdrawal can be retained for analysis or they would like their data to be destroyed instead.

The study was included in the National Institute of Health Research (NIHR) Clinical Research Network Portfolio (CPMS number: 45890) on September 25, 2020, and badged as NIHR Urgent Public Health Study on October 14, 2020. These measures facilitate the rapid mobilization of resources from NIHR and clinical research networks toward study dissemination and participant recruitment and help ensure that high-quality data can be collected on a timely basis (trial registration number: ISRCTN13953727).

### Dissemination Plan and Patient and Public Involvement

The RECAP-V0 template has already been disseminated nationally through CRNs facilitating the standardization of clinical records of patients with COVID-19. Its use has been encouraged through webinars and invited talks arranged by CRNs. Once the risk prediction tool has been developed and validated, we will seek endorsement for it to be incorporated into the electronic template to support clinical decision making when assessing patients with COVID-19. We expect to reach wide national and international dissemination of the RECAP-V1 risk prediction tool through submission to academic journals and international conferences.

Patient and public participation has been incorporated at different stages of the project. Patients were involved, along with primary care clinicians, in the qualitative study carried out to identify the set of elements to be included in the RECAP-V0 [15]. Once the RECAP-V1 tool has been developed and validated, public participation will be sought to coproduce project lay summaries, which will be valuable to disseminate the study findings to a wider audience.

## Discussion

The RECAP-V1 early warning score will, we anticipate, facilitate the stratification of the severity of patients with COVID-19 and their appropriate management and escalation of treatment. This study also promotes the standardization of assessment of patients with COVID-19, of collection of medical records, and record keeping thanks to the electronic templates developed, which can all have a positive impact in patients’ care, continuity, and safety [26]. Moreover, since November 2020, NHS England and NHS Improvement have led the establishment of the COVID Oximetry @home pathway, offered to patients with symptomatic COVID-19 who are aged 65 years or older or who are clinically extremely vulnerable to COVID-19 [27]. This service is being delivered by general practice, with referrals from NHS 111, CCAS, and hospital emergency departments, and involves an initial face-to-face or remote clinical assessment followed by monitoring of home oximetry readings for 14 days, to aid early recognition of deterioration. Items in the RECAP-V1 risk prediction tool in development are consistent with suggested clinical markers for triage on this pathway, and we anticipate that the tool will provide a unified quantitative risk score that will fit the monitoring needs of the service. Finally, we would like to emphasize the value of the study as an example of a digital clinical study, whose practice has been upheld by national research institutions on the basis of its cost-effectiveness and patient-centeredness due to the potential to recruit participants and collect large amounts of data with minimum inconvenience for the patient [28]. This is an example of leveraging the power of the NHS as a learning health system [29].

## Appendix

Multimedia Appendix 1 Peer review document.

Multimedia Appendix 2 Peer review comments.

## Abbreviations

CCG: Clinical Commissioning Group
COPI: Control of Patient Information
CRN: clinical research network
GP: general practitioner
HES: Hospital Episode Statistics
iCARE: Imperial’s Clinical Analytics, Research and Evaluation database
ICU: intensive care unit
NEWS2: National Early Warning Score
NHS: National Health Service
NIHR: National Institute of Health Research
ONS: Office of National Statistics
ORCHID: Oxford RCGP Clinical Informatics Digital Hub
RCGP: Royal College of General Practitioners
RECAP: Remote COVID-19 Assessment in Primary Care
RSC: Research and Surveillance Centre
SAP: statistical analysis plan
WSIC: Whole Systems Integrated Care
