# Supplementary material for: Predicting Risk of Hospital Admission in Patients With Suspected COVID-19 in a Community Setting: Protocol for Development and Validation of a Multivariate Risk Prediction Tool
Source: JMIR Res Protoc. 2021 May 25;10(5):e29072. doi: 10.2196/29072 (PMC8153031; doi:10.2196/29072)
Supplement: Multimedia Appendix 2 [file resprot_v10i5e29072_app2.pdf]

Imperial College London

NHS
Imperial College Healthcare
NHS Trust

Peer Review Office
Imperial College London and
Imperial College Healthcare NHS Trust
Room 221, Level 2, Medical School Building
Norfolk Place
London W2 1PG

| | | | |
|---|---|---|---|
| Project Title | RECAP (Remote Covid Assessment in Primary Care): a learning system approach to develop an early warning score for use by primary care practitioner | | |
| Chief Investigator | Professor Brendan Delaney | | |
| Reference number | **831** | Date of Issue | **19/05/2020** |

This certificate confirms that the research protocol for this project was submitted to the Imperial College Peer Review Office and that where applicable the correct peer review process was applied at an appropriate level of independence. For reviews obtained by the Peer Review Office, the questions asked of the reviewer were adequately addressed.

**This certificate is not a commentary on the scientific or methodological merit of the study.**

PEER REVIEW OFFICE ASSESSMENT AND ACTION:

☐ The project does not need peer review.

☐ The project has already undergone adequate peer review.

Details:

☐ The office obtained one appropriate and sufficiently independent peer review. The review should be supplied and considered with this certificate.

☒ The office obtained two appropriate and sufficiently independent peer reviews. The reviews should be supplied and considered with this certificate.

Dr Gule Hanid, Peer Review Officer
On Behalf of Director of the Peer Review Office

peerreviewoffice@imperial.ac.uk

Imperial College London

Reviewer 1 comments:

| Section A: Context | Yes | No |
|---|---|---|
| Is there evidence of adequate literature review? | YES | ☐ |
| Is the research a) worthwhile, b) innovative and c) timely? | YES | ☐ |
| **Please provide justification for your above answers:** | | |
| a) Very important research question due to Covid-19 pandemic, b) innovative design in several respects with one example being record linkage, c) clearly this is timely as we need answers during the current pandemic | | |

| Section B: Research design | | | | Yes | No |
|---|---|---|---|---|---|
| Is there a clearly defined, answerable question? | | | | YES | ☐ |
| Is the study design appropriate to the project? | | | | YES | ☐ |
| Is the proposed data analysis: a) described | Yes: YES | No: ☐ | b) appropriate | YES | ☐ |
| Are the methods likely to produce an answer to the above defined question? | | | | YES | ☐ |
| **Please provide justification for your above answers:** | | | | | |
| Clear research questions; appropriate design by a team that know what they are doing; clear and appropriate analysis plan; methods as described are likely to produce an answer to the defined question. | | | | | |

| Section C: Sampling | Yes | No |
|---|---|---|
| Is there justification for sample size? | YES | ☐ |
| Is the proposed sample representative of the target population? | YES | ☐ |
| Is there sufficient evidence to indicate that it will be possible to obtain the sample? | YES | ☐ |
| **Please provide justification for your above answers:** | | |
| Sample size justified, and the sample is likely to be reasonably representative of the target population. Given the strong drive for general practices to be involved in Covid-19 research to the exclusion of all other research then it is likely that the required sample will be obtained. | | |

| Section D: Clinical considerations |
|---|
| **Please discuss the clinical appropriateness of any drug, device or procedure administered to participants.** |
| Not applicable |
| **What are the potential burdens and risks for participants? (in the case of patients, burdens and risks above those expected in normal care)** |
| Though not likely to be any increased burdens or risks for participants |

| Section E: Practicalities | | | | Yes | No |
|---|---|---|---|---|---|
| Have appropriate resources been identified? | | | | Not sure | Not sure |
| Is the timescale realistic? | Yes: YES | No: ☐ | And likely to be met? | YES | ☐ |
| Are the researcher(s) and department able to carry out the project? | | | | YES | ☐ |

| **Please provide justification for your above answers:** |
|---|
| It is not clear whether this project has been funded yet. The timescales are realistic given the urgency of the task, and the fact (mentioned above) that general practitioners are likely to agree to participate. From what I know of the researchers and the departments, I am confident they would be able to carry out the research. |

| **Section F: Overall recommendation** |
|---|
| In terms of scientific quality and viability I recommend that this study is supported by the Research Ethics Committee. |

Reviewer 2 comments:

| Section A: Context | Yes | No |
|---|---|---|
| Is there evidence of adequate literature review? | X | ☐ |
| Is the research a) worthwhile, b) innovative and c) timely? | X | ☐ |
| **Please provide justification for your above answers:** | | |
| The authors comprehensively describe relevant prior work they have done as well as other publicly available early warning scores that currently exist to risk-stratify COVID-19 patients. There is a clear need to develop models to risk-stratify outpatients with COVID-19. | | |

| Section B: Research design | | | | Yes | No |
|---|---|---|---|---|---|
| Is there a clearly defined, answerable question? | | | | X | ☐ |
| Is the study design appropriate to the project? | | | | X | ☐ |
| Is the proposed data analysis: a) described | Yes: X | No: ☐ | b) appropriate | X | ☐ |
| Are the methods likely to produce an answer to the above defined question? | | | | X | ☐ |
| **Please provide justification for your above answers:** | | | | | |
| The early phase will allow for validation of an expert-derived score and a later phase will allow for data-drive development and validation of a logistic regression model-based score. | | | | | |

| Section C: Sampling | Yes | No |
|---|---|---|
| Is there justification for sample size? | X | ☐ |
| Is the proposed sample representative of the target population? | X | ☐ |
| Is there sufficient evidence to indicate that it will be possible to obtain the sample? | X | ☐ |
| **Please provide justification for your above answers:** | | |
| The sample size for each component is well-justified. | | |

| Section D: Clinical considerations |
|---|
| **Please discuss the clinical appropriateness of any drug, device or procedure administered to participants.** |

| |
|---|
| Not applicable. |
| **What are the potential burdens and risks for participants? (in the case of patients, burdens and risks above those expected in normal care)** |
| Main risks for model development appear to be breach of confidentiality. For the qualitative component, there may be some burden on clinicians' time to participate in semi-structured interviews and e-mail discussion but this appears manageable. |

| Section E: Practicalities | | | | Yes | No |
|---|---|---|---|---|---|
| Have appropriate resources been identified? | | | | X | ☐ |
| Is the timescale realistic? | Yes: ☐ | No: X | And likely to be met? | ☐ | X |
| Are the researcher(s) and department able to carry out the project? | | | | X | ☐ |
| **Please provide justification for your above answers:** | | | | | |
| Carrying out and analyzing 30 semi-structured interviews in 12 months is ambitious. But this timeline may still allow fro meaningful results from this study. | | | | | |

| **Section F: Overall recommendation** |
|---|
| Overall, this is a well-described study with a strong motivation and strong methodology in both the quantitative and qualitative aspects. |
