# Supplementary material for: Predicting Risk of Hospital Admission in Patients With Suspected COVID-19 in a Community Setting: Protocol for Development and Validation of a Multivariate Risk Prediction Tool
Source: JMIR Res Protoc. 2021 May 25;10(5):e29072. doi: 10.2196/29072 (PMC8153031; doi:10.2196/29072)
Supplement: Multimedia Appendix 1 [file resprot_v10i5e29072_app1.pdf]

Economic
and Social
Research Council

Professor Trisha Greenhalgh
University of Oxford
trish.greenhalgh@phc.ox.ac.uk (by e-mail)

12th May 2020

Dear Professor Greenhalgh

UKRI Ideas to Address COVID-19 grant call – Anonymised Peer Review Comments
Remote-by-Default Care in the COVID-19 Pandemic: addressing the micro-, meso-, and macro-level challenges of a radical new service model

In order to improve the transparency of the peer review process, applicants will receive Panel comments and/or consolidated comments from the Panel Meeting (if available). Although the attached feedback may not cover all the views expressed by the Panel, I hope that you will find them helpful in providing some background information on points raised by other reviewers in assessing this proposal.

I should emphasise that the Council takes no responsibility for the views expressed in the enclosed comments and does not necessarily endorse them. I am afraid that the ESRC is not able to enter into discussions arising from these comments and would be grateful if you could treat them as strictly confidential.

Introducer A
1. Strong fit to the call- develop new tools for COVID-19 symptom assessment to be used by call centre + GP surgeries, and examine their implementation across the sector. 2. High importance and urgency. 3. Highly experienced researchers, high costs of the research coming from high % of staff costs? 4. Some details of the research missing like how will you translate from the interviews with GPs and nurses to a set of breathlessness assessment questions? What is the comparison method? How will their symptom score be different from the existing NEWS2 score? What is the virtual in-residence researcher methods? Are a lot of these digital ethnography methods to be developed (no references)? They seem very time intensive, so strong participant burden. Rather than observation, the person being observed has to explain a lot of things. This has strong implications for ethical approval. 5. Very strong impact is likely. 6. But ethical approval status is doubtful, unless NIHR approves the burdensome methods

Introducer B
This proposal aims to provide new evidence and tools to improve the delivery of remote consultations in primary care.

Strengths: Strong team already rapidly producing Covid-relevant outputs (expert group at Oxford who specialise in digital innovation in health care) plus additional co-apps from Plymouth and Nuffield Trust. Clear application with highly practical deliverables (two new assessment tools, development of remote consultation service model, information to improve NHS infrastructure). Well connected to policy makers. Some leveraging of existing funding re-directed to this project. Qualitative elements (and outputs building on them) look particularly strong and the NASSS Appendix is very helpful.

Weaknesses: Expensive and not all costs well justified. Nuffield Trust consultancy costs look very high. Quantitative elements are less well described/convincing. There is a lack of detail about the RECAP early warning score tool or the

sample sizes for this element (Delphi panel of 50 GPs and analysis of 1,000 electronic records). The infrastructure strengthening element was more vague. It is presumed the whole proposal just relates to England.

Introducer C
1. The proposal meets the criteria in two ways
a. new research or innovation with a clear impact pathway that has the potential (within the period of the grant) to deliver a significant contribution to the understanding of, and response to, the COVID-19 pandemic and its impacts
b. gathers critical data and resources quickly for future research use
2. Importance of the project in the context of the national response to COVID-19
There has been a dramatic change in how primary care works as a result of the COVID-19 pandemic, with a massive shift to telephone/VC consultations and triage. It is not clear what impact this has had on patient care in terms of safety or acceptability nor in how clinicians have taken to it In addition it is not clear what symptoms and signs are important in terms of determining deterioration in COVID-19. This is a particular problem for those working remotely in the community. It is likely that current severity scoring systems are not ideally tailored to this purpose. Current ad hoc methods have no evidence base and urgent work is required to determine in terms of predicting deterioration which parameters are significant and at which weight in these should be applied. While it is likely others are pursuing these goals. I am not aware of active projects at the moment. There is a clear aim to produce pragmatic usable findings
3. Expertise and resources
In terms of the qualitative research this is an exceptionally strong team. I was less sure about the skills required to develop the predictive tool (possibly reflected in the lack of detail as to how this would be achieved) and wonder if some one with expertise in this area should be added to the team
4. Research and/or data collection plan
As above very clear description of the qualitative methods but disappointing description of the quantitative methods that would be employed. It is not clear how they arrived at the figure of 1000 to validate their model. Nor was there consideration given to alternative methods of developing the model. They have chosen to test a 'best guess' derived from a Delphi approach. A machine learning approach would be an alternative. This is not a 'killer' observation but could be remedied by adding someone with suitable expertise to the team.
5. Impact
Potentially a very high impact in terms of deriving a useful predictive test for GPs, but I would be happier with a better description of how this would be achieved. New knowledge about the impact of the huge switch to remote consulting (which may not be fully reversed post COVID-19) will help service planner and practitioners
6. Legal and ethical requirements
Unclear as to how the proposal complies with UKRI data requirements. Assume that this experienced team will be seeking the appropriate approvals.

Introducer D
This is a well written and very strong application from an excellent group of researchers who are leaders in the field of technological assessment in UK primary care.

The questions the assess are timely and highly important. The methods are appropriate and well developed. Greenhalgh's micro/meso/macro approach has worked well to address similar technological/implementation questions. Resources are reasonable.
The work should be funded subject to the comments below.

Minor comments:
It is not clear if the four sites represent general practices in which they personally are already working. This would be inappropriate.

Some practices are already very mature in their 'digital first' triage process, for example through eConsult or similar systems; are these to be selected or excluded?

eConsult, for example, gives extensive support on implementation of remote consulting – the applicants may want to refer to this if not already aware.

Practices are in many cases overwhelmed by COVID related work – are practices already signed up? It would be useful to have letters giving agreement in principle to take part.

Panel Feedback
The Panel agreed this was a highly important and extremely urgent study with clear potential to directly impact on the NHS, however there were some weaknesses. The digital ethnography methods were well described but there was a potential for participant burden. There was potential for ethical issues beyond those described in the proposal. Some aspects of the proposal were under-developed compared to the qualitative aspects, although it was noted that there were limitations of space of the form. Deliverables were considered very practical and worthwhile. It was noted that the team has an excellent track record and is well-connected to policy makers. It was agreed that leverage in existing funding for this project was a strength however the proposal was expensive and not all costs were well justified. There was a lack of detail in the RECAP early morning score tool and the infrastructure strengthening element was vague. The Panel noted that this proposal appeared to contain a significant proportion of NIHR remit, although social science made important contributions. Overall the Panel agreed this was an important proposal for better enabling virtual consultations going forward. It has subsequently been agreed that ESRC and NIHR will co-fund this grant, with ESRC being the lead funder (i.e. ESRC will issue the grant, and therefore be your primary point of contact).
